# Fungal isolates influence the quality of *Peucedanum praeruptorum* Dunn

**DOI:** 10.3389/fpls.2022.1011001

**Published:** 2022-10-24

**Authors:** Li Liu, Xuejun Wang, Shaotong Chen, Dong Liu, Cheng Song, Shanyong Yi, Fucheng Zhu, Wei Wang, Fang Wang, Guanglin Wang, Xiangwen Song, Bin Jia, Cunwu Chen, Huasheng Peng, Lanping Guo, Bangxing Han

**Affiliations:** ^1^ College of Biological and Pharmaceutical Engineering, West Anhui University, Lu’an, China; ^2^ Anhui Engineering Research Center for Eco-agriculture of Traditional Chinese Medicine, West Anhui University, Lu’an, China; ^3^ College of Life Science, South China Agricultural University, Guangzhou, China; ^4^ Analytical and Testing Center, West Anhui University, Lu’an, China; ^5^ School of Pharmacy, Anhui University of Chinese Medicine, Hefei, China; ^6^ State Key Laboratory Breeding Base of Dao-di Herbs, National Resource Center for Chinese Materia Medica, China Academy of Chinese Medical Sciences, Beijing, China

**Keywords:** *Peucedanum praeruptorum* Dunn, endophytes, rhizosphere microorganisms, secondary metabolites, field experiment

## Abstract

The symbiotic relationship between beneficial microorganisms and plants plays a vital role in natural and agricultural ecosystems. Although *Peucedanum praeruptorum* Dunn is widely distributed, its development is greatly limited by early bolting. The reason for early bolting in *P. praeruptorum* remains poorly characterized. We focus on the plant related microorganisms, including endophytes and rhizosphere microorganisms, by combining the traditional isolation and culture method with metagenomic sequencing technology. We found that the OTUs of endophytes and rhizosphere microorganisms showed a positive correlation in the whole growth stage of *P. praeruptorum*. Meanwhile, the community diversity of endophytic and rhizosphere fungi showed an opposite change trend, and bacteria showed a similar change trend. Besides, the microbial communities differed during the pre- and post-bolting stages of *P. praeruptorum*. Beneficial bacterial taxa, such as Pseudomonas and Burkholderia, and fungal taxa, such as Didymella and Fusarium, were abundant in the roots in the pre-bolting stage. Further, a strain belonging to *Didymella* was obtained by traditional culture and was found to contain praeruptorin A, praeruptorin B, praeruptorin E. In addition, we showed that the fungus could affect its effective components when it was inoculated into *P. praeruptorum*. This work provided a research reference for the similar biological characteristics of perennial one-time flowering plants, such as *Saposhnikovia divaricate*, *Angelica sinensis* and *Angelica dahurica*.

## Introduction

*Peucedanum praeruptorum* Dunn (“Qianhu” in Chinese) is a traditional Chinese medicinal plant famous for its dried roots and belongs to the *Peucedanum* genus in the Umbelliferae family. However, traditional views stated that bolting and flowering Qianhu could not be used as medicine. As a perennial and one-time flowering plant, Qianhu can grow in the field for 3–5 years before bolting and is widely cultivated by commercial growers due to its high medicinal value and large market demand. According to farmers, the bolting time of artificially planted Qianhu has gradually advanced and generally lasts for 1–2 years, leading to the insufficient accumulation of active ingredients and reduced medicinal value. After bolting, the area of secondary xylem increases, and its coumarin variant and content are reduced (Chen et al., 2019). To the best of our knowledge, the flowering mechanism remains unclear. Some scholars suspected the role of gene expression or environmental factors and thus explored differential genes in pre- and post-bolting. For example, one study showed that *Angelica sinensis* bolting and vegetative growth led to pollen germination and pollen tube growth (Yu et al., 2012). Emerging speculations indicated that programmed cell death and photoperiod regulation might be the causes of root lignification and lead to the migration of chemical components to the apical parts after bolting (Song et al., 2021).

Current research on Qianhu mainly focused on its chemical composition and pharmacological activity. This plant is generally used for the treatment of certain respiratory diseases, including anemopyretic cold and cough with phlegm accumulation (Li et al., 2019). Increasing evidence revealed that Qianhu also had a wide range of pharmacological effects, including anti-cancer (Zhang et al., 2021), anti-inflammatory (Lee et al., 2017), antitumor (Yuan et al., 2020), and antioxidant (Zhao et al., 2015). Various coumarins, especially angular-type pyranocoumarins including praeruptorin A, praeruptorin B and praeruptorin E, are the main active constituents that promote the efficacy of Qianhu (Chu et al., 2020). Praeruptorin A inhibits the migration and invasion of liver cancer cells and downregulates the expression of matrix metalloproteinase-1 by activating extracellular signal-regulated kinase (ERK) signaling pathways to inhibit the movement of liver cancer cells (Yu et al., 2021). Praeruptorin B decreases the migration and invasion ability of human renal carcinoma cells by suppressing the EGFR–MEK–ERK signaling pathway (Lin et al., 2020). Praeruptorin E suppresses protein extravasation in bronchoalveolar lavage fluid and attenuates myeloperoxidase activity and pathological lung changes (Yu P. J. et al., 2013). However, limited research has been reported on microorganisms, except for the two new strains *Streptomyces castaneus* (Zhou et al., 2017) and *Mumia xiangluensis* (Zhou et al., 2016) that were identified from Qianhu. Therefore, the endophytes in Qianhu could be regarded as rich resources and have great research value.

It has been proposed that microbiomes play an extremely important role in the growth of plants. Plant-related microorganisms colonize on the surface and inside of plants, and their source is possibly the soil around plants (Zarraonaindia et al., 2015). Endophytes are microorganisms that live specifically within the tissue of their host plants without causing apparent disease symptoms (Kui et al., 2021a). Endophytic microbiota possess a large number of unknown functional characteristics, such as influencing the timing of plant flowering (Lu et al., 2018), promoting plant growth (Kui et al., 2021b), suppressing phytopathogens (Lundberg et al., 2012), and helping plants withstand heat, salt, and drought (Alexander et al., 2020). Some endophytic microbiota and their host plants might have the same synthetic routes as some secondary metabolites and could produce similar or identical metabolites. For example, the fermentation extract of *Aspergillus fumigatus*, which was isolated from *Taxus*, contains paclitaxel (Kumar et al., 2019). The fermentation extract of *Fusarium solani*, which was isolated from *Camptotheca acuminata*, contains camptothecin (Ran et al., 2017). So, we think that the early bolting of Qianhu may be related to microorganisms.

Traditional culture techniques cannot characterize most soil microorganisms because they are not suitable for cultivating about 80%–99% of microbial species (Davis et al., 2005). Culture-independent high-throughput technology has greatly expanded the number of microbial species known to exist in plants and the surrounding environment (Yang et al., 2017). There was a technique to capture DNA fragments directly from samples using function-guided and sequencing-guided (phylogenetic markers) approaches, named metagenomics. With the development of sequencing technology, metagenomic research has penetrated humans, animals and plants, marine microbiomes, and complex soil microorganisms (Xu et al., 2018). By combining the traditional isolation and culture method with metagenomic sequencing technology, we can obtain comprehensive microbial classification information, isolate culturable microorganisms, and further identify their secondary metabolites.

In this study, we determined the relationship between endophytes and rhizosphere microorganisms in the whole growth stage of Qianhu and screened out the different strains. We employed the traditional culture method to obtain different fungi containing coumarins and analyzed their effects on the quality of Qianhu. The results provide evidence of an outstanding phenomenon: microorganisms play a vital role in the quality of Qianhu.

## Methods

### Sample collection and surface sterilization

The samples were collected from Donghekou Village (116.6567°E, 31.4044°N), Lu’an City, Anhui Province in China. This location has a subtropical monsoon climate with four distinct seasons: mild climate, moderate rainfall, sufficient light, and long frost-free period. The plant was identified by Professor Bangxing Han of West Anhui University. The soils within 2mm of the root surface were considered rhizosphere soils and extracted with a scalpel (DeAngelis et al., 2009). From May to December 2019, 3 individual Qianhu samples were collected every 15 days, and their root-associated samples were fractionated into two compartments: rhizosphere soils and roots. All scalpels and glassware used to collect roots and rhizosphere soils were disinfected with 75% ethanol to avoid cross contamination (Xia et al., 2021).

After the disinfection of all root samples, 100 μL of the last cleaning aliquot of water was inoculated into a plate containing potato dextrose agar medium to test the reliability of the method. The samples in the pre-bolting stage of Qianhu were labeled as CY1, CY2, CY3, CY4, and CY5, the corresponding rhizosphere soils were CYT1, CYT2, CYT3, CYT4, and CYT5, respectively. The samples in the post-bolting stage of Qianhu were labeled as CY6, CY7, CY8, and CY9, and the corresponding rhizosphere soils were CYT6, CYT7, CYT8, and CYT9, respectively. The flowering samples of Qianhu were labeled as CY10, CY11, and CY12, and the corresponding soils were CYT10, CYT11, and CYT12, respectively. The diversity and differences of microorganisms in the roots and rhizosphere soils of Qianhu were compared during the whole growth stage. CY1–CY5 were pre-bolting stages (CTQ), and CY6–CY12 were post-bolting stages (CTH).

### DNA extraction, sequencing, and metagenomic data analysis

DNA was extracted from each soil or root sample using E.Z.N.ATM Mag-Bind Soil DNA Kit (OMEGA, M5635-02, USA). The V3–V4 region of the 16S rRNA gene was used 341F (5′-CCTACGGGNGGCWGCAG-3′) and 805R (5′-GACTACHVGGGTATCTAATCC-3′) primers, while primers ITS1F (5′-CTTGGTCATTTAGAGGAAGTAA-3′) and ITS2R (5′-GCTGCGTTCTTCATCGATGC-3′) were used to amplify DNA fragment of internal transcribed spacer (ITS) region. PCR amplifications were conducted in a total volume of 30 μL containing 10-20 ng genomic DNA, 15 µL of 2×Taq Master Mix (Vazyme, P111-03, China), 1 μL of each forward/reverse primer (10 μM), and then made up the volume with ddH_2_O (nucleotide free). PCR was performed on a thermal instrument (BIO-RAD, T100TM Thermal Cycler, USA) as follows: 94°C for 3 min; 5 cycles at 94°C for 30 s, 45°C for 20 s, and 65°C for 30 s; and then 20 cycles at 94°C for 20 s, 55°C for 20 s, 72°C for 30 s, and final elongation at 72°C for 5 min. All products used as templates were subjected to second-step PCR by using Illumina-compatible primers. Samples were delivered to Sangon BioTech (Shanghai) for library construction using a universal Illumina adaptor and index. Sequencing was performed using the Illumina MiSeq system, according to the manufacturer’s instructions.

The obtained sequences were spliced and filtered by quality control to obtain effective data (Chen et al., 2020). Removing the non-amplified region sequence, correcting the error, and dividing the sequences into different OTUs according to their similarity were performed using Usearch. High-quality read data were aggregated into microbial OTUs using > 97% sequence identity (Almeida and De Martinis, 2019). Bacterial and fungal sequences were classified using RDP, Silva, and NCBI databases. Statistical data were analyzed with R, and Shannon and ACE diversity indexes were calculated by vegan software package in R. The differential abundance of microbial community composition was analyzed by linear discriminant analysis effect size (LEfSe). The features with significant differences related to the category of interest were detected by nonparametric factorial Kruskal–Wallis and rank tests. Consistency between the two groups was examined by Wilcoxon test. Finally, the impact of each differentially abundant feature was estimated by LEfSe using LDA (Segata et al., 2011).

### Construction of microbial correlation networks

The relationship between endophytic and rhizosphere microorganisms of Qianhu was determined through network analysis based on Spearman rank correlation. The top 100 with the highest abundance were selected for the OTU of each data set (endophytic fungi, endophytic bacteria, rhizosphere fungi, and rhizosphere bacteria). The Spearman correlation score was calculated by R, and visualization was conducted using Gephi 0.9.2. The nodes in the network represent OTUs, and the links connected between nodes represent close relationships (Shi et al., 2016).

### Isolation and identification of endophytic fungi from Qianhu roots

The fungi were isolated according to the metagenomic sequencing results. The roots were collected from the same plot, superficially disinfected, and cut into fragments of 2−3 mm arranged on a plate containing PDA medium with 0.1% penicillin–streptomycin solution and 0.1% sodium deoxycholate. The plate was placed at 28°C for 2–3 days in the dark until the root edge gradually generated hyphae. Sterilized sticks were used to isolate the hyphae, which were then inoculated on a new PDA medium. When the newly inoculated hyphae grew backward, the hyphae at the edge were extracted for culture and purified repeatedly until a pure culture was obtained. The strains were screened for preservation according to their color, size and growth time. Among them, “Q” represents the separation from the pre-bolting stage of Qianhu, “H” represents the separation from the post-bolting stage.

The purified endophytic fungus mycelia of Qianhu was scraped from the plate, and the DNA was extracted using a fungus extraction kit (Beijing Solarbio Science & Technology Co., Ltd.). PCR amplification was then carried out and visualized in 1% agarose gel to verify the amplification size. The sequencing results were compared using NCBI Blast. The homologous sequence with 100% similarity was preferred, followed by that with the highest similarity. Mega 6.0 software was used to construct the phylogenetic tree by the maximum likelihood method. The sequences were gathered in one branch with the closest genetic relationship and the highest similarity to determine the classification of the strains.

### Analysis of secondary metabolites in endophytic fungi

We refer to the extraction method in the Chinese Pharmacopoeia to extract the secondary metabolites of endophytic fungi (Committee for the Pharmacopoeia of P.R. China, 2020). Samples preparation: the same size of fungus block was extracted and transferred to a 500 mL Erlenmeyer flask containing 300 mL of potato dextrose broth (PDB) that was kept in an automatic rotary shaker at 28°C and 140 rpm for a week. After suction and filtration with the Brinell funnel, the culture broth and mycelium were separated. The culture broth was extracted with an equal volume of dichloromethane three times. The solvent was removed from the organic phase by rotary evaporation to yield the organic extract. The residue was then dissolved into methanol to obtain a test solution. According to the extraction method and chromatographic conditions in Chinese Pharmacopoeia, all the mycelium samples were dried in a 50°C oven and then grinded into powder. The sample powder was placed in an ultrasonic bath with methanol for 10 min. After cooling, the extracted solution was filtered and dried, and the residue was dissolved for HPLC analysis.

Chromatographic conditions: ZORBAX Eclipse Plus C_18_ column (150 mm × 4.6 mm, 5 μm) was applied, and the mobile phase consisted of water (A) and methanol (B) in gradient mode from 75 (B) at 0–30 min. The flow rate was 1.0 mL/min, the detection wavelength was 235 nm, and the injection volume was 10 μL. The column temperature was set at 30°C.

LC-MS qualitative analysis was performed using an Agilent 6545 Q-TOF tandem mass spectrometer. Separation was conducted using a ZORBAX Eclipse plus C_18_ column (2.1 ×100 mm, 1.8 μm, Agilent Technologies, USA). The eluent A was water, and the eluent B was methanol. The flow rate was 0.25 mL/min with a 2 μL injection volume, and the column temperature was set at 30°C. The gradient program was as follows: 0–3.00 min, 30%–75% B; 3.00–15.00 min, 75% B; 15.00–16.00 min, 75%–100% B;16.00–20.00 min, 100% B; 20.00–21.00 min,100%–30%; and 21.00–22.00 min, 30% B. The mass spectrometer parameters were set as follows: drying gas (N_2_) flow rate, 10 L/min; drying gas temperature, 320°C; nebulizer, 35 psig; sheath gas flow rate, 11 L/min; sheath gas temperature, 280°C; capillary voltage, 3500 V (positive mode); fragmentor, 175 V; skimmer, 65 V; and octopole RF peak, 750 V. The data were acquired under positive ion mode and 30 eV collision energy (CE). The full-scan mass range was 100–3000 Da. The reference ions of the calibration solution were at *m/z* [M+Na]^+^: 409.1263, 411.1420, 449.1577, and 451.1733.

### Inoculation of fungal suspension on Qianhu

Different fungi containing coumarins were selected for the inoculation test to explore the relationship between microorganisms and the quality of Qianhu. Qianhu seeds (from Ningguo City, Anhui Province, China) were disinfected and sown in the botanical garden of West Anhui University, and the layout of the trial plot was designed by a completely random design method (**Supplementary Figure 3**). 12 communities, each with 100 Qianhu seedlings, were established under normal field management. When the germinating seedlings grew to 5 cm high, the missing plants in the field were supplemented as needed.

The activated differential fungus was transferred to a 500 mL Erlenmeyer flask with 300 mL of PDB and kept in an automatic rotary shaker at 140 rpm and 28°C for 5 days. The mycelium was collected by suction filtration, and the stock solution was prepared after resuspension with sterile water and diluted 10, 20, and 40 times at high concentration (JH), medium concentration (JM), and low concentration (JL), respectively. Each concentration was analyzed using three biological replicates. In brief, 10 mL fungal suspension was poured near the root of each plant, and the same amount of sterile water was used for the control (CK).

### Endophytic colonization assessment

The unfolding Qianhu of each experimental plot was randomly collected with three plants in each plot, and then each group had nine repetitions. ITS high-throughput sequencing was carried out in accordance with the DNA extraction, sequencing steps. The obtained sequences were spliced and filtered by quality control to obtain effective data (Canarini et al., 2021). Removing the non-amplified region sequence, correcting the error, and diving the sequences into different OTUs according to their similarity were conducted by Usearch. Statistical analysis of biological information was performed on OTUs at 97% similar levels (Li et al., 2021). The fungal sequences were classified using RDP, Silva, and NCBI databases.

### Quantitative analysis of three coumarins in Qianhu experimental community

The bolting rate of each experimental plot was counted every 10 days. At harvest time, 3 individual Qianhu samples were collected in each plot, and then each group had nine repetitions. The pre- and post-bolting samples were collected to determine the contents of praeruptorin A, praeruptorin B and praeruptorin E.

Preparation of standard solution: appropriate amounts of praeruptorin A (National Institute for Food and Drug Control, purity > 99.4%), praeruptorin B (National Institute for Food and Drug Control, purity > 98.9%), and praeruptorin E (Shanghai Yuanye Biotechnology Co., Ltd., batch number: B20036, purity > 99.9%) were added to methanol to prepare praeruptorin A at 79.94 μg/mL, praeruptorin B at 86.88 μg/mL, and praeruptorin E at 58.69 μg/mL.

Preparation of test solution: Qianhu samples before and after bolting in each experimental plot were collected at harvest time and analyzed using three biological replicates. All samples were dried in the shade and then grinded into powder, which were then passed through an 80-mesh sieve. Afterward, 0.2 g of sample powder was placed in an ultrasonic bath with 25 mL of methanol for 30 min. After cooling, the extracted solution was filtered for HPLC analysis (Hu et al., 2020).

Chromatographic conditions: ZORBAX Eclipse Plus C_18_ column (150 mm×4.6 mm, 5 μm) was applied. The mobile phase consisted of water (A) and methanol (B) in gradient mode from 50% to 100% (B) at 0–40 min. The flow rate was 1.0 mL/min, the detection wavelength was 321 nm, and the injection volume was 10 μL. The column temperature was set at 30°C.

SPSS 26.0 software was used for one-way ANOVA. The data were expressed as mean ± SD. The data before and after bolting were processed by a multi-component paired test. P<0.05 suggested statistically significant difference.

## Results

### Temporal dynamics of microbial interkingdom

**Figure 1:**
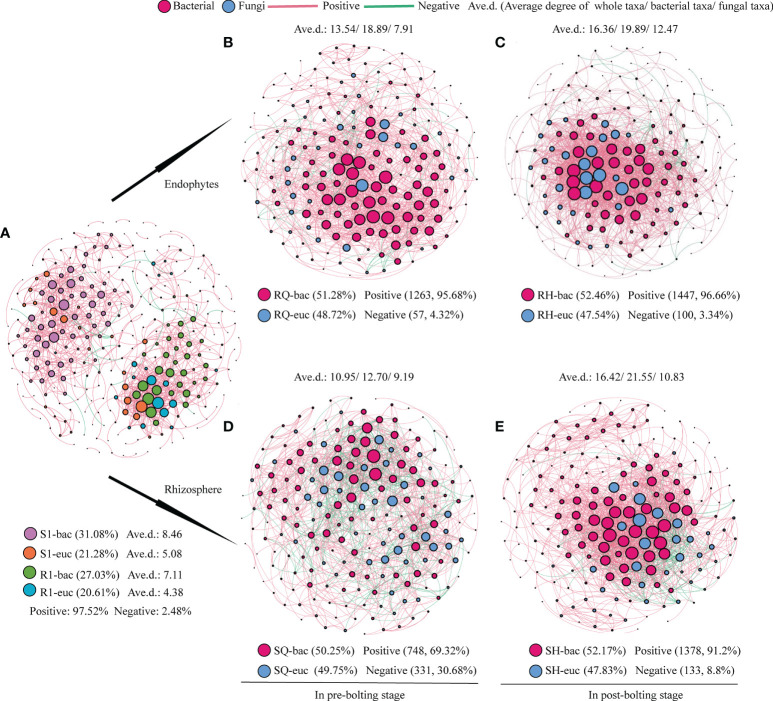
Co-occurrence networks of endophytes and rhizosphere microbial community. **(A)** endophytes and rhizosphere microbial community in the whole growth stage; **(B)** endophytic bacteria and fungi in pre-bolting stage; **(C)** endophytic bacteria and fungi in post-bolting stage; **(D)** rhizosphere bacteria and fungi in pre-bolting stage; and **(E)** rhizosphere bacteria and fungi in post-bolting stage.

Co-occurrence network analysis was performed to assess the relationship between the endophyte and rhizosphere microorganism OTUs of Qianhu. A significant Pearson rank correlation was observed in microbial diversity (P < 0.05). Regardless of endophytes or rhizosphere microorganisms, the network connectivity of fungi was less than that of bacteria during the whole growth stage of Qianhu, and a positive correlation was found between endophytes and rhizosphere microorganisms (**Figure 1A**). The network pattern of microorganisms changed in the pre- and post-bolting stages. The network connectivity of endophytic bacteria in Qianhu increased from 13.54% in pre-bolting stage to 16.36% in the post-bolting stage, in which endophytic bacteria increased from 18.89% to 19.89% and endophytic fungi increased from 7.91% to 12.47%. The network connectivity of the rhizosphere microorganisms increased from 10.95% in the pre-bolting stage to 16.42% in the post-bolting stage, including rhizosphere bacteria from 12.70% to 21.55% and rhizosphere fungi from 9.19% to 10.83%. In the whole growth stage of Qianhu, endophytic fungi and endophytic bacteria mainly showed a positive correlation. However, the positive correlation ratio between rhizosphere fungi and bacteria increased from 69.32% in the pre-bolting stage to 91.20% in the post-bolting stage (**Figures 1B–E**).

**Figure 2:**
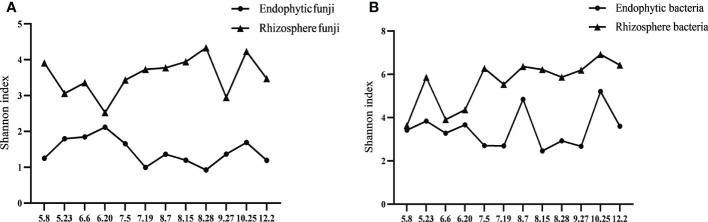
Shannon index line chart of Qianhu in the whole growth stage. **(A)** Fungi and **(B)** Bacterial.

Analysis of the alpha diversity index showed that whether fungi or bacteria, the diversity of rhizosphere microorganisms was significantly higher than that of endophytes. The change trend of endophytic and rhizosphere fungi in the majority of Qianhu growth periods showed the opposite trend and was similar only in the last stage possibly because the soil no longer needed to provide nutrition for plants (**Figure 2A**). The change trend of endophytic bacteria in Qianhu and rhizosphere bacteria was extremely complex. A similar trend was observed from May 8 (CY1) to June 20 (CY4), the opposite from June 20 (CY4) to July 19 (CY6), the same from July 19 (CY6) to August 15 (CY8), the opposite from August 15 (CY8) to September 27 (CY10), and the same from September 27 (CY10) to December 2 (CY12) (**Figure 2B**). June 20–July 19 was the stage when Qianhu was about to bolt, and August 15–September 27 was the stage when Qianhu was about to blossom. During these times, rhizosphere bacteria were in short supply, and opposite changes were observed.

### Diversity and community assembly of bacterial and fungal microbiomes across different plant developmental stages

**Figure 3:**
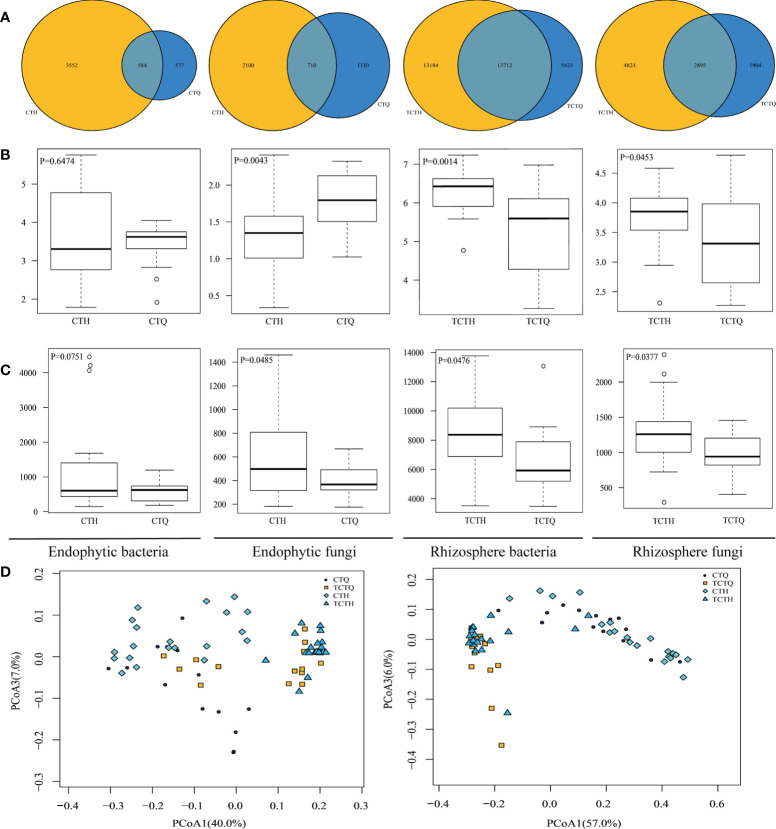
Differences of Qianhu in pre- and post-bolting periods. **(A)** Number of OTUs shared among different Qianhu samples. The number in the shaded overlap area indicated the total number of OTUs shared between sample types. **(B)** Sample diversity measurements of endophytic and rhizosphere samples. Species diversity was estimated according to the Shannon index. **(C)** Species community richness was estimated according to the ACE index. **(D)** PCoA analysis between roots and soil samples based on weighted unifrac distance. Bacteria can be found on the left and fungi on the right.

The whole metagenomic analysis showed significant differences in pre- and post-bolting stages. The Venn diagram showed the number of shared and unique OTUs. Overlaps were observed in differentially abundant OTUs in the rhizosphere compartments, indicating a large difference in endophytes but small difference of rhizosphere microorganisms in Qianhu (**Figure 3A**). Shannon’s index was higher in the rhizosphere soils than in the corresponding roots. In addition, the endophytic fungi in the pre-bolting stage possessed relatively high microbiome diversity and low community richness, and endophytic bacteria showed no difference. Therefore, we speculated that endophytic fungi played a more important role in the development of Qianhu than endophytic bacteria. The rhizosphere microorganisms in the pre-bolting stage possessed relatively low microbiome diversity and community richness (**Figures 3B, C**). The principal coordinate analysis (PCoA) of the microbial communities from Qianhu using the weighted unifrac distance metrics suggested that the rhizosphere compartments had separated across PCoA1 (**Figure 3D**, 40% in bacteria and 57% in fungi). However, the three principal components cannot separate the samples in pre- and post-bolting stages.

**Figure 4:**
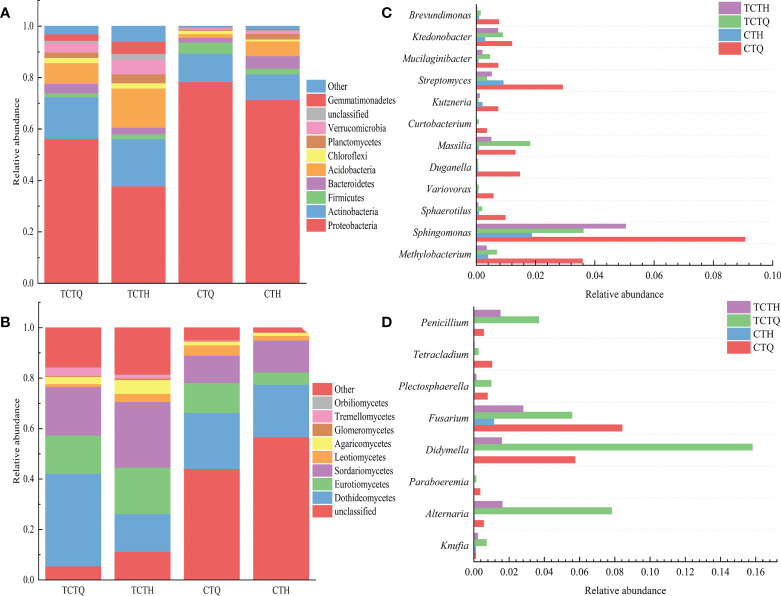
Taxonomic composed of bacterial and fungal microbiomes. **(A)** Bacterial community composition in the pre-and post-bolting stages of Qianhu; **(B)** Fungal community composition; **(C)** Comparison of bacterial communities that decreased after bolting as screened by LEfSe analysis; **(D)** Comparison of fungal communities.

The endophytic bacterial community was divided into systematic types and consisted of 31 phyla. Among which, Proteobacteria was the most abundant phylum, accounting for > 70% in roots and > 30% in soil (**Figure 4A**). The endophytic fungal community was classified into 12 phyla and 53 classes, in which Dothideomycetes was the most abundant class irrespective of varieties. Actinobacteria in the rhizosphere soil were more abundant in the post-bolting stage (18.30%) than in the pre-bolting stage (16.24%); the opposite pattern was observed in the roots. Regardless of endophytes or rhizosphere microorganisms, abundance decreased for Proteobacteria (from 78.40% to 71.31% in endophytes and from 56.26% to 37.85% in rhizosphere microorganisms) and increased for Acidobacteria (from 1.51% to 5.72% in endophytes and from 8.09% to 15.27% in rhizosphere microorganisms) after bolting (**Figure 4A**). In addition, the relative abundance of Dothideomycetes in the endophytic and rhizosphere decreased (from 22.23% to 20.81% in endophytes and from 36.73% to 14.96% in rhizosphere microorganisms), and that of Sordariomycetes in the endophytic and rhizosphere increased (from 10.75% to 12.66% in endophytes and from 19.25% to 26.04% in rhizosphere microorganisms). Eurotiomycetes in plant compartments were more abundant at the pre-bolting stage (11.88%) than at the post-bolting stage (4.83%); the opposite pattern was observed in the roots (**Figure 4B**).

LEfSe analysis revealed that a plethora of microorganisms changed before and after bolting. A decrease in *Ktedonobacter*, *Streptomyces*, *Kutzneria*, *Curtobacterium*, *Duganella*, *Variovorax*, and the other 11 genera, and an increase in *Barnesiella*, *Actinophytocola*, *Mycobacterium*, *Terriglobus*, *Edaphobacter*, and the other 21 genera were observed after bolting in plant compartments (**Supplementary Figure 1A**). A decrease in *Paraboeremia*, *Berkleasmium*, *Knufia*, *Penicillium*, and the other 12 genera and an increase in *Berkleasmium* and *Coniochaeta* occurred after bolting (**Supplementary Figure 1B**). A decrease in *Rhodococcus*, *Serratia*, *Pantoea*, and other 8 genera and an increase in *Amycolatopsis*, *Leifsonia*, *Aridibacter*, and the other 18 genera were observed after bolting in rhizosphere compartments (**Supplementary Figure 1C**). A decrease in *Lectera*, *Humicola*, *Filobasidium*, and other 16 genera and an increase in *Coniosporium*, *Chaetomella*, *Coniochaeta*, and the other 25 genera occurred after bolting in the rhizosphere compartments (**Supplementary Figure 1D**). *Mucilaginibacter*, *Massilia*, *Methylobacterium*, *Alternaria*, *Fusarium*, and *Didymella* decreased after bolting but neither in the root nor rhizosphere soil (**Figures 4C, D**).

### Isolation and screening of coumarin-producing fungi

**Figure 5:**
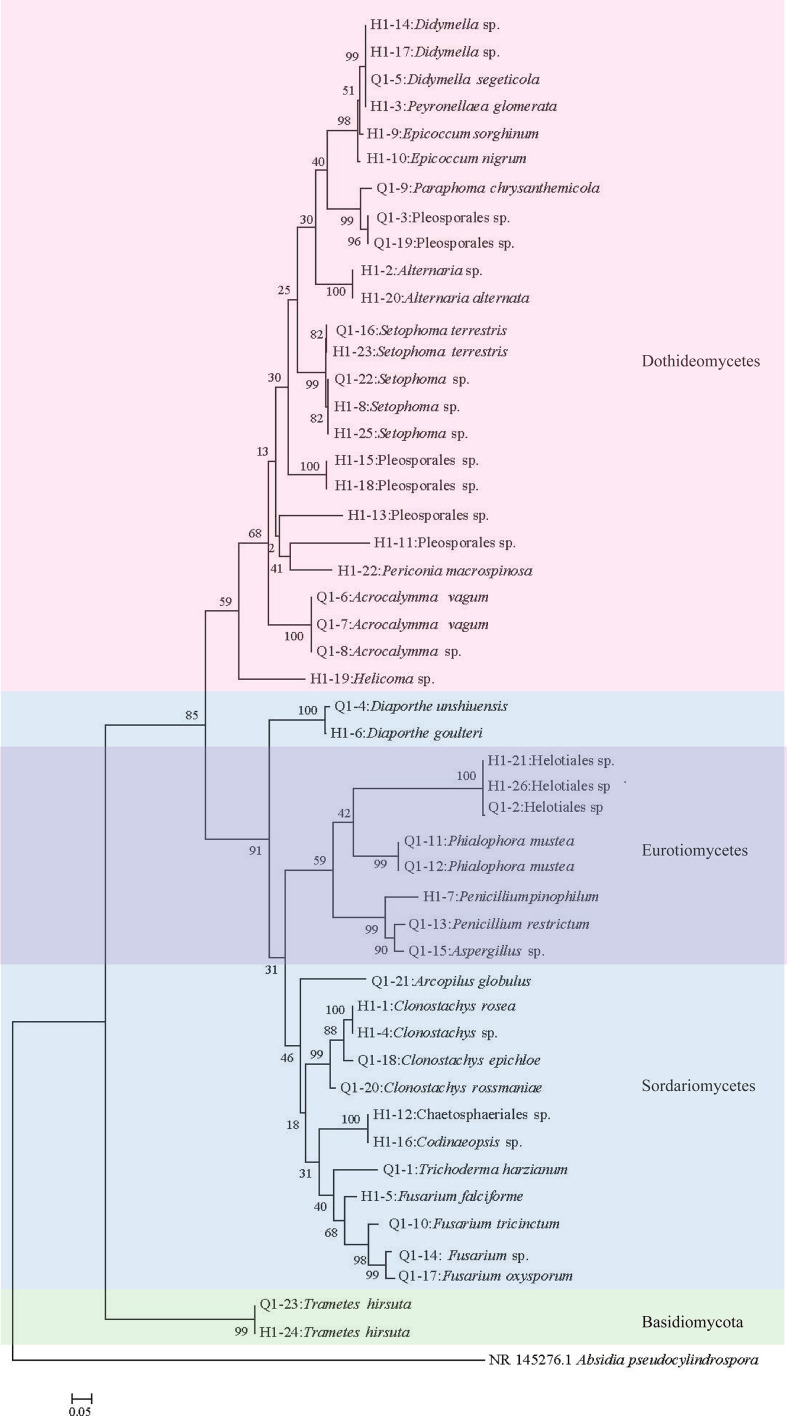
Phylogenetic tree of endophytic fungi in Qianhu. Each color corresponds to a class.

Considering that the diversity of endophytic fungi showed significant differences in the pre- and post-bolting stages of Qianhu, this study focused on isolating endophytic fungi. 49 strains of endophytic fungi with different morphologies were obtained, including 23 in pre-bolting and 26 in post-bolting. Blast search on Genbank revealed that among the 49 strains, 27 were identified to the species level, 12 to the genus level, and 10 to the order level, thus producing a total of 23 species, 18 genera, 16 families, 9 orders, 4 classes, and 2 phyla (**Figure 5**). Among the 23 strains in pre-bolting stage, 16 were identified to the species level, 4 to the genus level, and 3 to the order level, thus producing a total of 17 species, 12 genera, 12 families, 7 orders, and 4 classes. Among the 26 strains in post-bolting stage, 8 were identified to the genus level, 11 to the species level, and 7 to the order level, thus producing a total of 15 species, 11 genera, 10 families, 7 orders, and 4 classes (**Figure 5**). Although the number of endophytic fungi isolated from the pre-bolting stage of Qianhu was less than that from the post-bolting stage, the diversity in the pre-bolting stage was richer than that in the post-bolting stage.

The coumarins of endophytic fungi were screened by HPLC and LC/MS. Several coumarins were found in the mycelial extract of 17 strains. Four coumarins (praeruptorin A, praeruptorin B, praeruptorin E and peucedanocoumarin I) were observed in 14 strains. Among which, 10 strains were isolated before bolting, and only 4 strains were isolated after bolting. In addition, 3 strains isolated before bolting contained three coumarins (praeruptorin A, praeruptorin B and peucedanocoumarin I or praeruptorin A, praeruptorin B and praeruptorin E). The 17 strains belonged to 13 species, 11 genera, and two orders (**Table 1**). Furthermore, Q1-5, Q1-11, Q1-12, Q1-14, and Q1-19 were the dominant bacteria. Whether as an endophyte or rhizosphere microorganism, Q1-5 (*Didymella segeticola*) was significantly reduced after bolting (P<0.05).

**Table 1:** Coumarins in endophytic fungus of Qianhu.

Number	Strains	praeruptorin A		praeruptorin B		praeruptorin E		peucedanocoumarin I	
		Retention time/min	Experimental Mass (*m/z*)	Retention time/min	Experimental Mass (*m/z*)	Retention time/min	Experimental Mass (*m/z*)	Retention time/min	Experimental Mass (*m/z*)
Standard		6.199	409.1263	8.976	449.1577	10.795	451.1733	6.827	411.1420
Q1-1	*Trichoderma harzianum*	6.237	409.1257	9.031	449.1569	10.916	451.1724	6.799	411.1412
Q1-2	Helotiales sp.	6.253	409.1260	9.064	449.1572	10.816	451.1726	6.799	411.1414
Q1-4	*Diaporthe unshiuensis*	6.234	409.1256	9.028	449.1570	10.780	451.1726	6.796	411.1412
Q1-5	*Didymella segeticola*	6.245	409.1261	9.055	449.1571	10.808	451.1729	/	/
Q1-6	*Acrocalymma vagum*	6.241	409.1256	9.035	449.1567	10.771	451.1725	6.786	411.1414
Q1-7	*Acrocalymma vagum*	6.246	409.1257	9.023	449.1565	10.775	451.1725	6.775	411.1415
Q1-11	*Phialophora mustea*	6.231	409.1258	/	/	10.760	451.1724	6.776	411.1414
Q1-12	*Phialophora mustea*	6.237	409.1258	9.047	449.1569	10.949	451.1724	6.799	411.1400
Q1-13	*Penicillium restrictum*	6.257	409.1258	9.034	449.1569	10.786	451.1725	6.802	411.1415
Q1-14	*Fusarium* sp.	6.237	409.1259	9.031	449.1569	10.767	451.1726	6.782	411.1407
Q1-16	*Setophoma terrestris*	6.242	409.1259	9.036	449.1570	10.788	451.1725	6.788	411.1416
Q1-19	Pleosporales sp.	6.236	409.1257	/	/	10.749	451.1725	6.798	411.1411
Q1-20	*Clonostachys rossmaniae*	6.257	409.1262	9.051	449.1571	10.803	451.1728	6.802	411.1414
H1-2	*Alternaria* sp.	6.252	409.1258	9.013	449.1568	10.930	451.1725	6.781	411.1416
H1-8	*Setophoma* sp.	6.237	409.1259	8.998	449.1569	10.899	451.1725	6.783	411.1413
H1-20	*Alternaria alternata*	6.257	409.1258	9.018	449.1570	10.770	451.1725	6.786	411.1413
H1-22	*Periconiamacros pinosa*	6.244	409.1261	9.005	449.1568	10.757	451.1726	6.789	411.1415

### Effects of *D. segeticola* on the quality of Qianhu

**Figure 6:**
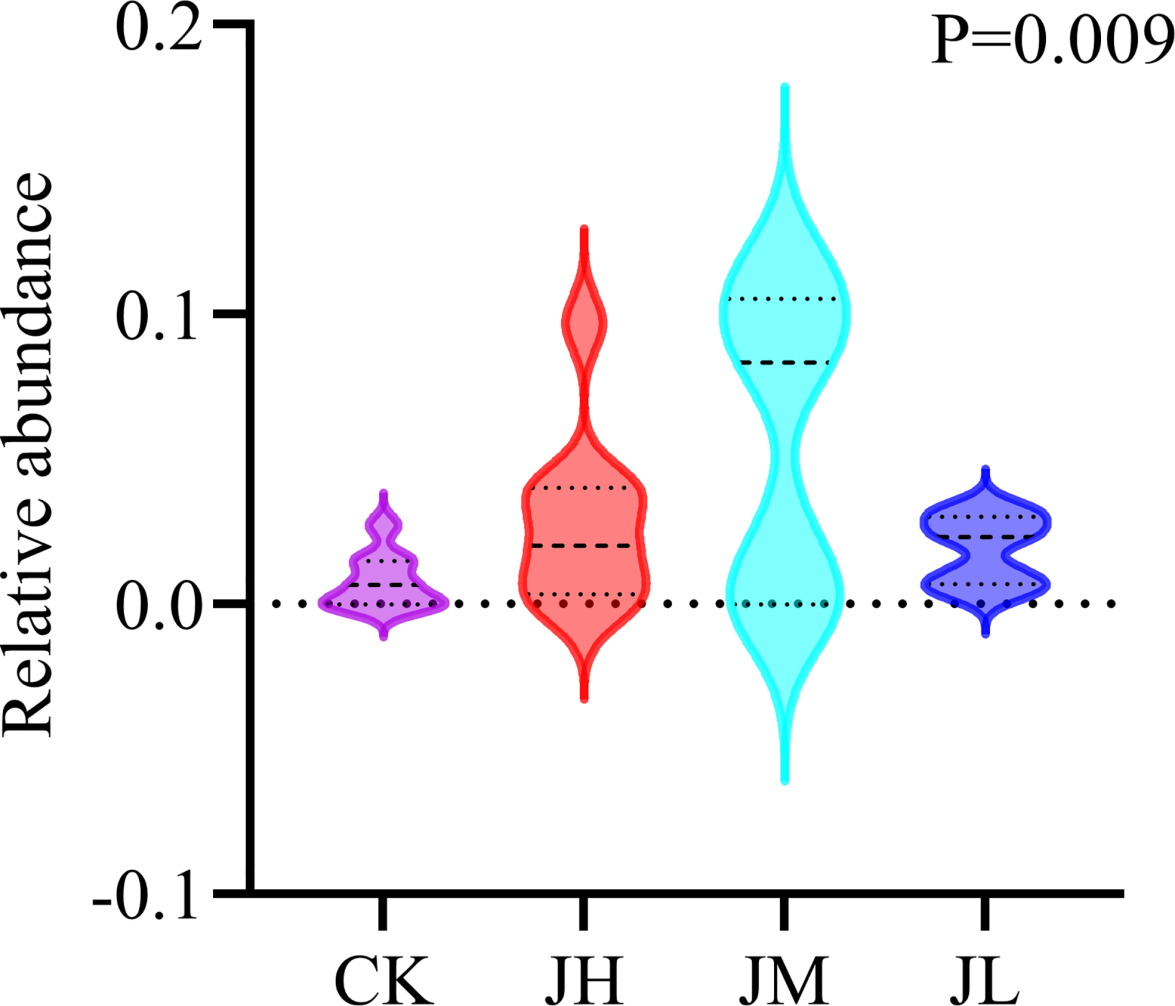
Analyzed of different fungal abundances in different treatment groups.

We hypothesized that one of the reasons for the quality decline of Qianhu after bolting is the reduced abundance of *D. segeticola* containing coumarins. For hypothesis testing, Qianhu seedlings were watered with different concentrations of fungal suspension. The results showed that the abundance of *D. segeticola* in treated Qianhu plants was significantly higher than that in the CK, especially in those watered at medium concentration. This finding indicated the fungus successfully colonized in Qianhu (**Figure 6**). The bolting rate of Qianhu in each experimental area was counted every 10 days until no new bolting Qianhu was observed. In the early growth stage of Qianhu, the bolting time in the CK was early and stable. Although there was no significant differences between the 4 groups, the bolting rate of low concentration experimental plot was always the lowest during the whole growth period (**Table 2**).

**Table 2:** Bolting rate of Qianhu in different periods.

Group	Bolting rate/%						
	2021.8.27	2021.9.6	2021.9.16	2021.9.27	2021.10.11	2021.10.21	2021.11.8
CK	6.13 ± 3.19	7.81 ± 5.04	7.84 ± 5.07	8.45 ± 5.59	9.59 ± 5.71	9.76 ± 5.85	10.09 ± 5.28
JH	5.01 ± 2.64	6.09 ± 4.27	6.15 ± 3.51	6.48 ± 3.15	7.54 ± 2.55	8.40 ± 2.87	9.12 ± 2.85
JM	3.00 ± 1.73	5.37 ± 0.55	8.08 ± 0.08	8.16 ± 0.00	10.83 ± 1.76	11.18 ± 2.28	11.18 ± 2.28
JL	1.72 ± 0.62	2.45 ± 1.51	3.95 ± 1.15	4.67 ± 1.63	5.50 ± 1.19	5.92 ± 1.55	5.92 ± 1.55

±, used to indicate the precision of an approximation.

**Table 3** shows the coumarin contents in the pre- and post-bolting stages of Qianhu. In the pre-bolting stage, the content of praeruptorin A under medium concentration treatment (0.806 ± 0.348) decreased compared with that in the CK (1.312 ± 0.446, P<0.05), the content of praeruptorin B under low concentration treatment (0.112 ± 0.045) decreased comparing with that in the CK (0.188 ± 0.087, P<0.05), and the content of praeruptorin E in medium (0.187 ± 0.057) and high concentration (0.236 ± 0.109) treatments decreased comparing with that in the CK (0.349 ± 0.156, P<0.05). However, no significant difference was observed in the total content of the three coumarins. After bolting, the contents of the three coumarins decreased significantly. In addition, the content of praeruptorin A increased under low concentration treatment (0.620 ± 0.168) comparing with that in the CK (0.433 ± 0.156, P<0.05). Overall, the total content of the three coumarins under low concentration treatment (0.883 ± 0.252) increased comparing with that of the CK (0.740 ± 0.251, P<0.05). These results showed that the application of fungal suspension at low concentration was helpful to delay the sudden decline of coumarins after the bolting of Qianhu.

**Table 3:** Content of coumarins in different groups of Qianhu (n=9, mean ± SD).

Group	Praeruptorin A (%)		Praeruptorin B (%)		Praeruptorin E (%)		Total (%)	
	Before bolting	After bolting	Before bolting	After bolting	Before bolting	After bolting	Before bolting	After bolting
CK	1.312 ± 0.446a	0.433 ± 0.156a	0.188 ± 0.087a	0.155 ± 0.184a	0.349 ± 0.156a	0.152 ± 0.071a	1.849 ± 0.540a	0.740 ± 0.251a
JL	1.412 ± 0.343a	0.620 ± 0.168b	0.112 ± 0.045b	0.076 ± 0.046a	0.344 ± 0.100a	0.187 ± 0.082a	1.840 ± 0.348a	0.883 ± 0.252b
JM	0.806 ± 0.348b	0.392 ± 0.229a	0.289 ± 0.248a	0.072 ± 0.066a	0.187 ± 0.057b	0.095 ± 0.039b	1.833 ± 0.347a	0.559 ± 0.245a
JH	1.137 ± 0.413a	0.468 ± 0.124a	0.229 ± 0.228a	0.084 ± 0.091a	0.236 ± 0.109b	0.120 ± 0.033b	1.826 ± 0.406a	0.672 ± 0.149a

Different lowercase letters in the same column indicate significant differences (P<0.05). ±, used to indicate the precision of an approximation.

## Discussion

### Plant developmental stages drive the differentiation in the ecological role of Qianhu-related microbiomes

Plant roots are surrounded by complex microbial communities either living in the soil or attached to the rhizosphere (Romdhane et al., 2021). The related microbiomes of terrestrial plants are divided into three spaces, namely rhizoplane compartment, rhizosphere compartment, and endophytic compartment (Lundberg et al., 2012; Edwards et al., 2015; Luo et al., 2017; Xiong et al., 2021). The diversity of fungal and bacterial communities is generally reduced from soil to flowers (Sauer et al., 2021). Moreover, the richness of endophytic bacteria and fungi in plants is lower than that in rhizosphere (Xia et al., 2021). Comparable observations were also found in Qianhu. Our results suggested that the endophytic community of Qianhu root has lower diversity or richness than the microbial community of its rhizosphere. These findings further showed that plant endophytes are a subset of rhizosphere microorganisms.

From cooperation to competition, positive or negative interactions occur between microorganisms. In cooperative interaction, microorganisms can divide labor, and some of them specialize in tasks beneficial to other individuals. Meanwhile, competition between microorganisms can be direct or indirect. The direct effect is to rob the resources needed for the survival of other microorganisms, and the indirect effect is to synthesize substances that inhibit the growth of other microorganisms (Romdhane et al., 2021). The host can selectively regulate microbial interactions to meet its needs during plant growth (Xiong et al., 2021). Microorganisms compete with many other microbes in the rhizosphere for nutrients and space, and these microbes affect them to some extent through complex interaction networks (Segata et al., 2011). The present correlation study revealed that the OTUs of rhizosphere microorganisms and endophytes were positively correlated in the whole growth stage of Qianhu, and this relationship was also found in shrub plants (Zuo et al., 2021). Moreover, the positive correlation between rhizosphere fungi and bacteria increased in the post-bolting stage. These findings suggested that the relationship between the rhizosphere bacteria and fungi of Qianhu gradually changes from competition to cooperation. It could be explained by during long-term co-evolution, complex interaction and cross-talk between endophytes and their hosts, such as signal exchange and horizontal gene transmission (Tan and Zou, 2001), have occurred to jointly cope with biotic and abiotic stresses.

The diversity of endophytic fungi and rhizosphere fungi showed the opposite trend. This may be due to the rapid growth of bacteria and slow growth of fungi. When plants recruit a number of bacteria and fungi from the rhizosphere, the rapid reproduction of rhizosphere bacteria makes them get timely replenished. However, the slow reproduction of rhizosphere fungi makes them recover slowly, which shows the opposite trend. Meanwhile, the change trend of endophytic bacteria and rhizosphere bacteria were similar most of the time and only the opposite during bolting and flowering. A possible explanation is that the plant is in the stage of rapid growth, and the rhizosphere microbial community is in short supply. The formation of fungi in rhizosphere soils and roots is affected by random changes, and their response to environmental factors differ from that of bacteria (Trivedi et al., 2021).

Robinson (Robinson et al., 2015). believes that roots are a favorable place for endophyte colonization. The rhizosphere contains a variety of microbial communities that play a key role in plant growth and reproduction (Lu et al., 2018). Investigation of microbial diversity in herbal medicine revealed the rhizosphere components of different plant species (such as phylum and family level) change at the phylogenetic level (for example, *Avena fatua*, tomato, and *Arabidopsis thaliana*) under similar field conditions, regardless of their geographical source locations (Panke-Buisse et al., 2015; de Souza et al., 2016, de Souza et al., 2021, Xiong et al., 2021). A comprehensive study of the endophytic microbial community gathered during the maturation of Qianhu established an endophytic dominant phylum composing of Proteobacteria and Ascomycota. Symbiotic members of the dominant microbiota were selectively recruited and enriched in parallel and adapted to life on plant tissues. Comparable observations were also found in studies of citrus (Xu et al., 2018) and tomato (Lee et al., 2019).

Plant development stages have an important effect on the composition and function of microbial communities. And microbial communities also play a crucial role in different stages of plant development. This study provides insights into the endophytic microbiota of Qianhu, which has not been described before. The root and soil microbial communities in the early stage were different from those in the bolting stage. Qianhu entered reproductive growth after bolting, and the OTU of endophytic and rhizosphere microorganisms increased significantly. The abundance of endophytic genera changed with plant development. *Sphingomonas*, *Variovorax*, *Gemmatimonadete*, *Rhizobacter*, and *Acidobacterium* can be used as candidate microorganisms to reflect soil fertility and plant health (Liu et al., 2021). Actinomycetes can produce nitrogen-containing organic compounds in soil and even decompose soil humus (Zhang et al., 2019). Acidobacteria generally exist in places where nutrition is scarce. After bolting, the abundance of Proteobacteria decreased and that of Acidobacteria increased, indicating the low nutrition in rhizosphere soil after bolting. *Pseudomonas* can alleviate the effects of heavy metal stress on the rhizosphere (Luo et al., 2017), and some fungal strains of *Trichoderma* and *Fusarium* have biocontrol activity (Lee et al., 2019). At the early bolting stage of Qianhu, abundant beneficial microbial groups can be found in the microbial community, such as *Pseudomonas*, *Burkholderia*, *Serratia*, *Pantoea*, and *Massilia*. However, their abundance decreased after bolting. It is similar with abundant beneficial bacterial taxa in maize microbiomes in the early stage, but saprophytic fungi in the late stage (Xiong et al., 2021).

### Endophytic fungi could function as micro factories with coumarins in Qianhu

Plant secondary metabolites have a wide range of potential applications in pharmaceutical, food, and cosmetic industries by providing new chemicals and compounds. However, the direct isolation of such compounds from plants has led to excessive harvesting and biodiversity loss, which threatens the extinction of several medicinal plant species (Mishra et al., 2021). Endophytic fungi live in plant tissues, play an important role in plant growth and development, and produce unique bioactive secondary metabolites. Several previous phytochemical have found that endophytic fungi and their host plants may synthesize some secondary metabolites in the same way, resulting in similar metabolites (Nicoletti et al., 2014). For example, the *Phialocephala fortinii* strain obtained from *Rhodiola* plants can produce salidroside and tyrosol compounds (Cui et al., 2016). In the present study, 49 strains of endophytic fungi were isolated from Qianhu, and their mycelia were extracted after fermentation. The results revealed 17 strains of endophytic fungi with the ability to produce coumarins, including *Trichoderma*, Helotiales sp., *Diaporthe*, *Didymella*, *Acrocalymma*, *Phialophora*, *Penicillium*, *Fusarium*, *Setophoma*, Pleosporales sp., *Clonostachys*, and *Alternaria*. Among which, *Didymella*, *Phialophora*, *Fusarium*, and pleosporales sp. were the dominant fungi in Qianhu. These results suggested that endophytic fungi play a vital role in the synthesis of coumarins in Qianhu. Although this is the first study to reveal that endophytic fungi can produce coumarins in Qianhu, previously published works already showed that *Fusarium*, *Alternaria*, and *Penicillium* have the ability to produce compounds similar to their host plants. A *Fusarium* sp. isolated from *Fritillaria unibracteata* can produce gallic acid, rutin, and other compounds (Pan et al., 2017). *Fusarium solani* isolated from *Camptotheca acuminata* can produce camptothecin (Ran et al., 2017). *Alternaria alternata* isolated from *Capsicum annuum* can produce capsaicin (Devari et al., 2014). *Penicillium* sp. isolated from *Sonneratia apetala* can produce polyketones (Liu et al., 2016). In the present study, few endophytic fungi were isolated in the pre-bolting stage of Qianhu, but these species were rich and diverse. The number of endophytic fungi that can produce coumarins of Qianhu was significantly higher in the pre-bolting stage than that in the post-bolting stage. This phenomenon may also be one of the reasons for the proportion changes of the main coumarins in Qianhu in pre- and post-bolting (Yu N. J. et al., 2013). Follow-up studies will focus on quantifying the coumarins of endophytic fungi and then screening out the best fermentation process for industrial production.

### Endophytic microorganisms can influence the quality of Qianhu

Endophytes can asymptotically colonize plants and exert important biological, physical, and chemical effects on the biosphere (Liu et al., 2017). Plant-associated microbiomes protect against pathogens, improve growth by enhancing plant nutrition (Averill et al., 2019), and help plants withstand environmental perturbations, such as abnormal variation in temperature, drought, and salinity related to climate (Mendes et al., 2011; Fitzpatrick et al., 2018). Host plants may attract beneficial microorganisms by regulating plant microbial-related signaling pathways (Gao et al., 2021). The inoculation of arsenic-contaminated rice with beneficial microorganisms can reduce the accumulation of arsenic and promote the growth of rice (Aw et al., 2020). Beneficial microorganisms can also reduce the adverse effects of pests by increasing proline yield by enhancing antioxidant enzyme activity, stimulating protease and polyphenol oxidase activity and providing additional phenols, protein, and chlorophyll to promote plant yield (Bano & Muqarab, 2017). Studies have used multigeneration experimental systems to screen soil microorganisms that induce the advance or delay of host flowering time and found that fungi may regulate flowering time, change extracellular enzyme activity, and improve reproductive biomass (Panke-Buisse et al., 2015). The early bolting of Qianhu has largely been studied, but the role of plant microbiota, particularly rhizosphere microbiota, has not been considered. *Didymella* is a pathogen of tea leaf spots (Huang et al., 2021). In the present study, this fungus was found to be the different fungus in the pre- and post-bolting stages of Qianhu. In addition, different concentrations of *D. segeticola* suspension had different effects on Qianhu. Early bolting of Qianhu was alleviated. Although the secondary metabolites of the strain contained praeruptorin A, praeruptorin B, and praeruptorin E, the contents of these coumarins in Qianhu were reduced after inoculation and were almost as low as those in the CK. In summary, the low concentration of *D. segeticola* suspension can reduce the bolting rate of Qianhu on the premise of ensuring the content of its effective components. Additional extensive studies are needed to decisively determine the interactive functions that occur in Qianhu and their endophytes.

## Data availability statement

The original contributions presented in the study are publicly available. TS sequence data can be accessed from the following link: https://www.ncbi.nlm.nih.gov/sra/PRJNA826278; 16S sequence data can be accessed from the following link: https://www.ncbi.nlm.nih.gov/sra/PRJNA827944; Sequencing data after inoculation can be accessed from the following link: https://www.ncbi.nlm.nih.gov/sra/PRJNA827982.

## Author contributions

BH and LG designed the project. LL, XW, SC, FZ, WW, FW, GW, XS, and BJ performed the experiments. CS, SY, CC, and HP analyzed the data. LL and DL wrote the manuscript. All authors contributed to the article and approved the submitted version.

## Funding

This research was supported by China Agriculture Research System of MOF and MARA(CARS-21), Excellent Youth Foundation of Anhui Natural Science (1808085J17) and the Major Increase or Decrease Project at the Central Level (2060302), Anhui Provincial Academic Funding Program for Top Disciplines (Specialties) in Colleges and Universities (gxbjZD2020083).

## Acknowledgments

We thank Ying Yang and Tingting Hou for their assistance with irrigated fungal suspension, and Houjun Yao for providing the experimental plot.

## Conflict of interest

The authors declare that the research was conducted in the absence of any commercial or financial relationships that could be construed as a potential conflict of interest.

## Publisher’s note

All claims expressed in this article are solely those of the authors and do not necessarily represent those of their affiliated organizations, or those of the publisher, the editors and the reviewers. Any product that may be evaluated in this article, or claim that may be made by its manufacturer, is not guaranteed or endorsed by the publisher.
