# Supplementary material for: Fungal isolates influence the quality of Peucedanum praeruptorum Dunn
Source: Front Plant Sci. 2022 Oct 24;13:1011001. doi: 10.3389/fpls.2022.1011001 (PMC9638934; doi:10.3389/fpls.2022.1011001)
Supplement: Supplementary file 1 [file DataSheet_1.zip › Supplementary Material.docx]

Supplementary Material


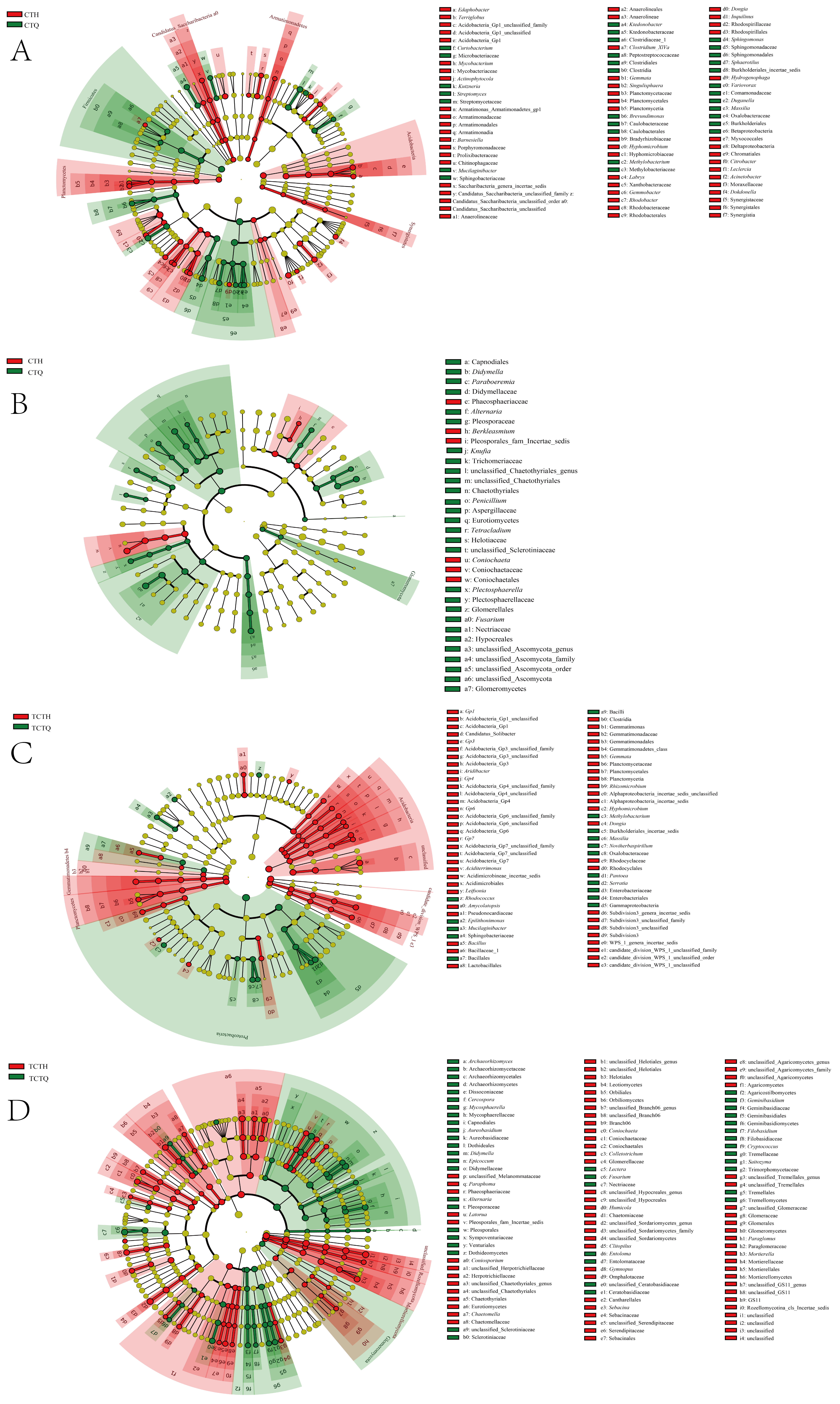


**Supplementary Figure 1** Cladograms of LEfSe showing bacterial and fungal taxa with significant differences in relative abundance between pre-bolting and post-bolting stage of Qianhu. The filled circles from inside to outside indicate the taxonomic levels with phylum, class, order, family, genus, and species. Circles or nodes shown in color corresponding to different plant species represented a significantly more abundant group. Yellow circles indicate species with no significant differences in relative abundance. **(A)** endophytic bacteria; **(B)** endophytic fungi; **(C)** rhizosphere bacteria; **(D)** rhizosphere fungi.


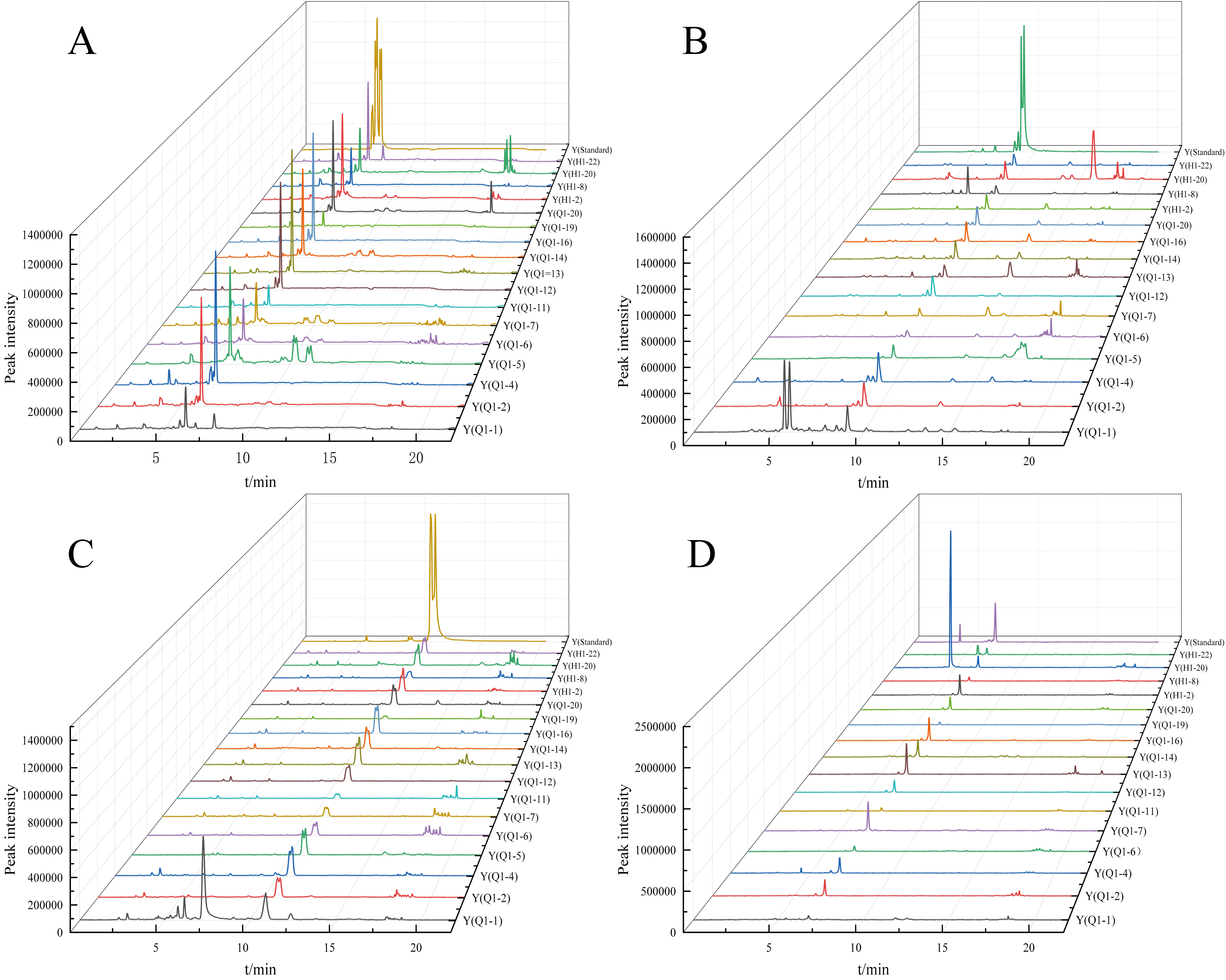


**Supplementary Figure 2** Extraction ion chromatogram of Coumarins of endophytic fungi. The mycelial samples had the same peak as the standard at the same time. **(A)**Praeruptorin A; **(B)**Praeruptorin B; **(C)**Praeruptorin E; **(D)**Peucedanocoumarin I.

JH-1

JL-2

JM-3

CK-2

JH-3

JL-1

CK-1

JM-1

CK-3

JH-2

JM-2

JL-3

保护行

保护行

保护行

保护行

50cm

50cm

50cm

2m

40cm

50cm

50cm

3m


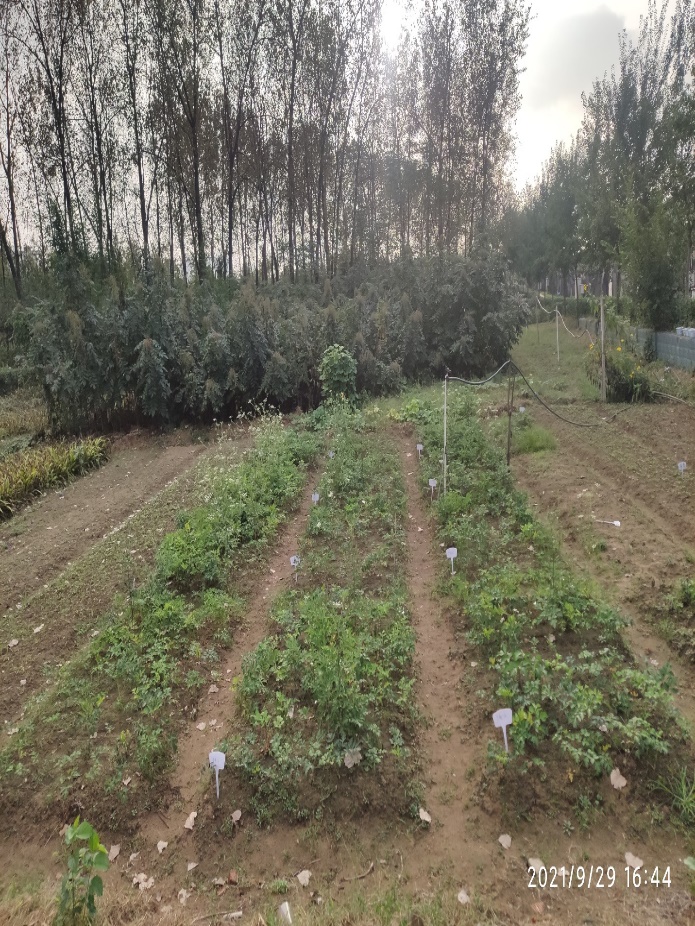


**Supplementary Figure 3** Layout of experiment plot. Note: JH: High concentration area; JM: Mediumconcentration area; JL: Low concentration area

**Supplementary** **Table 1** Bolting rate of Qianhu during the different period

| Test plot | Bolting rate | | | | | | |
| --- | --- | --- | --- | --- | --- | --- | --- |
|  | 2021.8.27 | 2021.9.6 | 2021.9.16 | 2021.9.27 | 2021.10.11 | 2021.10.21 | 2021.11.8 |
| CK-1 | 6.00% | 11.11% | 11.00% | 12.00% | 13.00% | 13.00% | 13.00% |
| CK-2 | 3.00% | 2.00% | 2.00% | 2.00% | 3.00% | 3.00% | 4.00% |
| CK-3 | 9.38% | 10.31% | 10.53% | 11.34% | 12.77% | 13.27% | 13.27% |
| JH-1 | 4.04% | 4.00% | 4.00% | 5.00% | 5.00% | 6.00% | 6.00% |
| JH-2 | 3.00% | 3.26% | 4.26% | 4.35% | 7.53% | 7.61% | 9.78% |
| JH-3 | 8.00% | 11.00% | 10.20% | 10.10% | 10.10% | 11.58% | 11.58% |
| JM-1 | 5.00% | 6.00% | 8.08% | 8.16% | 9.00% | 9.00% | 9.00% |
| JM-2 | 2.00% | 5.10% | 8.16% | 8.16% | 12.50% | 13.54% | 13.54% |
| JM-3 | 2.00% | 5.00% | 8.00% | 8.16% | 11.00% | 11.00% | 11.00% |
| JL-1 | 1.01% | 1.03% | 3.13% | 3.13% | 4.17% | 4.17% | 4.17% |
| JL-2 | 2.04% | 4.04% | 5.26% | 6.38% | 6.45% | 6.45% | 6.45% |
| JL-3 | 2.11% | 2.27% | 3.45% | 4.49% | 5.88% | 7.14% | 7.14% |
